# Management of alveolar‐pleural fistula secondary to invasive pulmonary aspergillosis with bronchial occlusion using a combination of Endobronchial Watanabe Spigot and N‐butyl‐2‐cyanoacrylate: A case report

**DOI:** 10.1002/rcr2.1163

**Published:** 2023-05-16

**Authors:** Shinya Tsukamoto, Kazuma Nagata, Keisuke Tomii

**Affiliations:** ^1^ Department of Respiratory Medicine Kobe City Medical Centre General Hospital Kobe Japan

**Keywords:** alveolar‐pleural fistula, aspergillus empyema, Endobronchial Watanabe spigot, invasive pulmonary aspergillosis, N‐butyl‐2‐cyanoacrylate

## Abstract

Alveolar‐pleural fistulas that do not improve with thoracic drainage can be conservatively treated via endobronchial occlusion and pleurodesis, among other options. However, for inoperable cases, the treatment strategy to be followed, in the event that conventional conservative management fails, is unclear. Herein, we report a case of alveolar‐pleural fistula managed by bronchial occlusion using a combination of Endobronchial Watanabe Spigot (EWS) and N‐butyl‐2‐cyanoacrylate (NBCA). A 79‐year‐old man on prednisolone for interstitial pneumonia with autoimmune features was diagnosed with invasive pulmonary aspergillosis and Aspergillus pyothorax infection. He was administered voriconazole; however, a pneumothorax developed and did not improve with thoracic drainage. Bronchial occlusion with EWS failed due to spigot migration. However, a combination of EWS with NBCA could control the alveolar‐pleural fistula. Thus, an EWS and NBCA combination may help prevent EWS migration, providing another option for patients who are unfit for surgery.

## Graphical abstract

Alveolar‐pleural and bronchopleural fistulas that do not improve with thoracic drainage in patients who are unfit for surgery can be successfully managed by endobronchial occlusion and pleurodesis. However, the strategy to be followed in case of failure in conventional conservative management is unclear. We report a case of alveolar‐pleural fistula managed by bronchial occlusion using a combination of Endobronchial Watanabe Spigot and N‐butyl‐2‐cyanoacrylate.

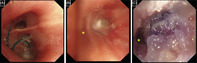

## INTRODUCTION

Alveolar‐pleural or bronchopleural fistulas may occur after pulmonary aspergillosis or necrotizing pneumonia; refractory cases without improvement with thoracic drainage have been reported.^1^ Conservative management includes endobronchial occlusion or pleurodesis.^2^ However, the strategy after conventional conservative treatment failure for surgically unfit patients is unclear. Herein, we report a case of conservative management of alveolar–pleural fistula following invasive pulmonary aspergillosis refractory to conventional conservative management.

## CASE REPORT

**FIGURE 1:**
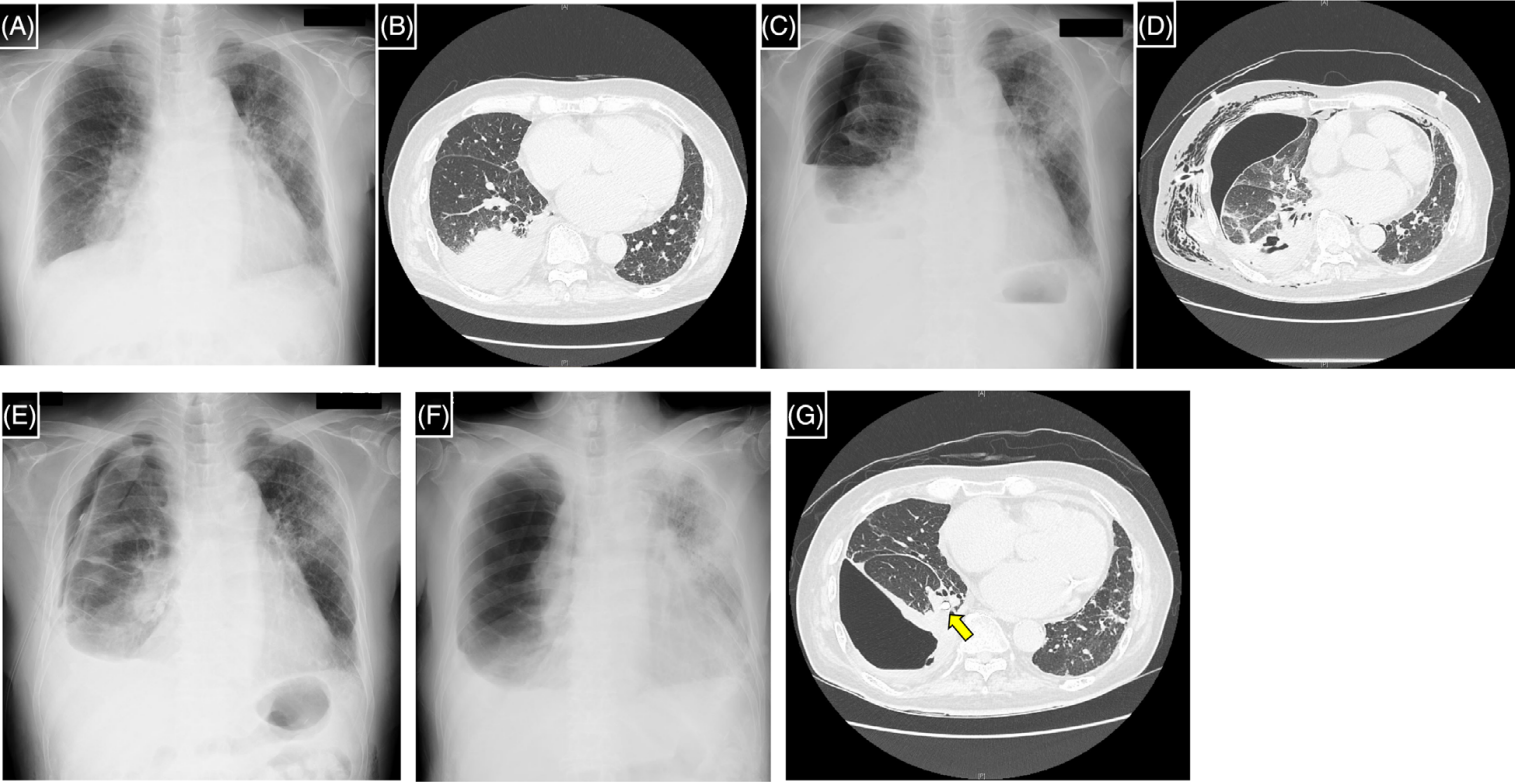
Chest radiograph and computed tomography (CT) of the patient performed at the time of admission (A, B). Chest radiograph performed when the pneumothorax occurred (C). CT examination, performed after pneumothorax was observed on chest radiograph, showing the dilated right B9/10 which appeared to connect with the thoracic cavity (D). Chest radiograph performed after the bronchial occlusion was treated using a combination of Endobronchial Watanabe Spigot and N‐butyl‐2‐cyanoacrylate (E). Chest radiograph and CT performed when right‐sided pneumothorax re‐emerged and left‐sided ground‐grass opacity worsened (F, G). The yellow arrow shows the remaining EWS in the right B9 (G).

**FIGURE 2:**
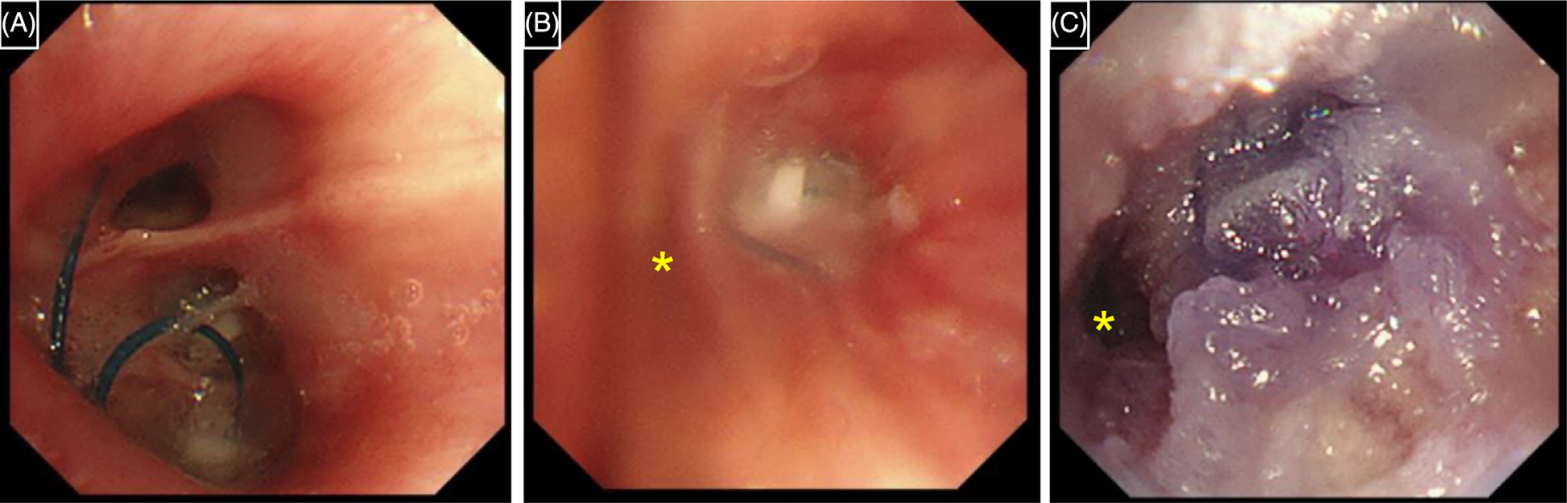
Bronchoscopic findings during bronchial occlusion using a combination of Endobronchial Watanabe Spigot (EWS) and N‐butyl‐2‐cyanoacrylate (NBCA). Findings after insertion of 6 mm EWS into the right B10 (A), after insertion of 7 mm EWS above the right B9/10 (B), and after depositing NBCA (C). The yellow asterisk shows the right B7.

A 79‐year‐old man was diagnosed with idiopathic interstitial pneumonia with anti‐double strand DNA antibody, that is, interstitial pneumonia with autoimmune features, 1 month prior. The initial administration of methylprednisolone (60 mg/day) was subsequently changed to prednisolone (40 mg/day). Three days before admission he experienced dyspnoea. Laboratory findings showed elevated C‐reactive protein (CRP) (19.41 mg/dL). Chest radiograph and computed tomography (CT) revealed consolidation in the lower lobe of the right lung along with right pleural effusion (Figure 1A, B). β‐glucan (234 pg/mL) and Aspergillus antigen (3.4) were identified, suggesting invasive pulmonary aspergillosis (IPA). Voriconazole was initiated on day 8 of hospitalization. Thoracentesis was performed on day 11. Aspergillus antigen in the pleural fluid was positive, and *Aspergillus fumigatus* was subsequently detected in culture, indicating Aspergillus empyema secondary to IPA. Despite the good clinical course, the patient's oxygenation saturation level worsened transiently on day 17. Chest radiography demonstrated right‐sided pneumothorax (Figure 1C). Thoracic drainage was initiated; however, the right lung collapsed regardless of suction, which may have been caused by an alveolar‐pleural fistula in the right S9/10 secondary to IPA. Considering the worsening performance status and protracted wound healing due to prednisolone, he was deemed unfit for surgery. Subsequently, we decided to occlude the bronchi using Endobronchial Watanabe Spigots (EWS) on day 31. During bronchoscopy, we first tried to assess the responsible bronchus for the air leak using the ballooning occlusion test; however, the leak stopped owing to the patient's supine position. We then inserted a 6 mm EWS into the right B9 and B10 segments respectively based on the CT image that shows that the dilated right B9/10 appeared to connect with the thoracic cavity (Figure 1D). The right lung expanded on that day but re‐collapsed the following day. Chest radiograph showed migration of EWS in the right B10 segment. On day 36, we re‐inserted a 6 mm EWS into the right B10 and a 7 mm EWS above B9/10, but they also failed in a similar manner. We then attempted blood patch pleurodesis which failed as well. On day 46, we planned bronchial occlusion with a combination of EWS and N‐butyl‐2‐cyanoacrylate (NBCA) to prevent EWS migration. First, we inserted a 6 mm EWS into the right B10 (Figure 2A) and deposited 1 mL of a 1:1 mixture of Lipiodol and NBCA. We then inserted a 7 mm EWS above the right B9/10 (Figure 2B) and deposited 1 mL of the same mixture (Figure 2C). The right lung expanded (Figure 1E) and thoracic drainage was completed on day 50. The EWS and NBCA were extracted on day 55, and right‐sided pneumothorax re‐emerged; however, additional thoracic drainage was unnecessary because the right lung remained expanded to some extent. Right lung hyperlucency was apparent and judged as a result of the pneumothorax (Figure 1F, G). Moreover, ground‐glass opacity in the left lung and CRP levels worsened (Figure 1F). We concluded that IPA was deteriorating and added micafungin to voriconazole on day 60. CRP levels improved and did not worsen after the completion of micafungin treatment on day 81. Right lung expansion and left lung ground‐glass opacity did not worsen, indicating he was stable. The patient was transferred to rehabilitation on day 87.

## DISCUSSION

We report a case of alveolar‐pleural fistula secondary to invasive pulmonary aspergillosis, which was managed by bronchial occlusion using a combination of EWS and NBCA after the failure of bronchial occlusion with EWS and blood patch pleurodesis. To the best of our knowledge, this is the first report on the use of EWS and NBCA to prevent EWS migration after the failure of EWS alone due to migration. Moreover, this is only the second report of bronchial occlusion using a combination of EWS and NBCA for alveolar‐pleural fistula. Successful management of alveolar‐pleural fistulas using conservative treatments, particularly bronchial occlusion and pleurodesis, has been reported^2^; however, the treatment strategies after the failure of existing conservative treatment for patients unfit for surgery remain unclear. This case report could provide a solution to this problem.

A combination of EWS and NBCA may help avert EWS migration. Bronchial occlusion with EWS is a conservative treatment option for alveolar‐pleural fistulas. A previous study reported that bronchial occlusion with EWS allowed drain removal in 85.7% of cases, but EWS dropout occurred in 23% of all cases.^3^ NBCA, the primary component of tissue adhesives, has been used for bronchial occlusion.^4^ When EWS and NBCA are used together, NBCA may fill the gap between the EWS and bronchial wall and act as an adhesive, preventing the EWS from dropping. This combination of EWS and NBCA may be a promising option when bronchial occlusion with EWS fails due to EWS migration.

We believe that ascended conservative treatment strategies for alveolar‐pleural fistulas may prevent surgery. Patients like ours with IPA are probably unfit for surgery due to background pulmonary disease and immunocompromised status. A previous study on prognosis after surgical treatment for secondary spontaneous pneumothorax caused by underlying diseases reported a significantly poor prognosis in patients with interstitial pneumonia.^5^ Therefore, establishing a conservative treatment strategy to avoid surgery is important.

We report the case of an alveolar‐pleural fistula secondary to invasive pulmonary aspergillosis, which was managed by bronchial occlusion with combined EWS and NBCA use after failure of EWS alone due to migration. Bronchial occlusion for management of alveolar‐pleural fistulas can be performed using multiple substances. The optimal combination for bronchial occlusion warrants further investigation.

## AUTHOR CONTRIBUTIONS

Shinya Tsukamoto drafted this manuscript. Kazuma Nagata and Keisuke Tomii revised the manuscript. All the authors approved the final manuscript.

## CONFLICT OF INTEREST STATEMENT

The authors declare no conflict of interests.

## ETHICS STATEMENT

The authors declare that appropriate written informed consent was obtained for the publication of this manuscript and the accompanying images.

## Data Availability

The data that support the findings of this study are available from the corresponding author upon reasonable request.
